# How best to determine causative pathogens of pneumonia

**DOI:** 10.1186/s41479-016-0004-z

**Published:** 2016-04-12

**Authors:** David R. Murdoch

**Affiliations:** Department of Pathology, University of Otago, Christchurch, P.O. Box 4345, Christchurch, 8140 New Zealand

**Keywords:** Pneumonia, Diagnostics, Aetiology, Polymerase chain reaction, PCR

## Abstract

The biggest recent development in pneumonia diagnostics has been the increased availability and use of nucleic acid detection assays, although this change has brought with it new challenges about the interpretation of positive results. Recognition of the existence of the lung microbiome has challenged the traditional views of pneumonia pathogenesis and may provide the opportunity for new diagnostic tools that are focused on more than just detection of specific known pathogens.

In 2009, some colleagues and I wrote a commentary on pneumonia diagnostics [1]. This is a challenging area. Historically, even with the best of methods, we have been unable to define a causative pathogen in a significant proportion of pneumonia episodes, especially in children. The impact of suboptimal diagnostics extends beyond individual patient care, also limiting our ability to assess interventions such as vaccines. Timed to coincide with the first World Pneumonia Day, our commentary tried to encourage a fresh look at pneumonia diagnostics. How has the world changed in the past seven years?

In 2009, we were still reliant on traditional diagnostics based on microscopy and culture—methods that had been around for decades. There had been modest advances in antigen detection methods, limited mainly to pneumococcal disease in adults and Legionnaires’ disease. Nucleic acid detection tests (NATs), such as polymerase chain reaction (PCR), had been established for all major pneumonia pathogens and were the single biggest development in the area, although widespread uptake by diagnostic laboratories had been slow. We highlighted several issues that needed addressing in order to move forward. The inability to obtain good quality specimens from the lower respiratory tract was a fundamental problem. Many diagnostic methods, such as blood culture, had poor diagnostic sensitivity, while the assessment of diagnostic accuracy was made challenging due to the lack of suitable comparator (“gold”) standards. Importantly, assigning causation was a struggle. Many pneumonia pathogens can also be asymptomatic colonisers of the upper respiratory tract, so distinguishing innocent bystanders from true pathogens can be challenging when detected in clinical specimens from non-sterile sites, such as expectorated sputum. We emphasised the need to look beyond technology, and the importance of assessing diagnostic performance within clinical and epidemiologic contexts. We were also hopeful for innovative new approaches.

So, what does the situation look like in 2016? A major change for diagnostic laboratories over the past seven years has been the increased availability of commercial NATs for respiratory pathogens. There are now many choices, typically in multiplex format, and the range continues to expand. Indeed, debate has started about whether these large multiplex panels should be subjected to restricted use in order to ensure appropriate use [2]. Furthermore, the successful deployment around the world of the Xpert® MTB/RIF assay (Cepheid, United States of America) for tuberculosis diagnostics has demonstrated the potential use of NATs beyond well-equipped laboratories and into resource-limited settings [3]. Despite their increased availability, NATs do not yet have clearly established roles for some major pneumonia pathogens. In particular, the inability to reliably distinguish *Streptococcus pneumoniae* carriage from disease when testing respiratory specimens is a major limitation, although setting thresholds based on bacterial load has shown promise in adults with pneumonia [4]. While used in some surveillance programmes [5], PCR testing of blood specimens for *S. pneumoniae* is not an established test for diagnosing pneumococcal pneumonia in individual patients due to uncertainty about diagnostic accuracy across different populations.

Therefore, in the context of pneumonia, NATs are still mainly useful for detection of non-colonising bacteria, such as *Legionella* species [6] and *Mycobacterium tuberculosis* [3], and for respiratory viruses. Even then, case-control studies have demonstrated the need for caution with the interpretation of respiratory virus test results, as positive results have been found to be as common in healthy controls as among pneumonia patients, especially for viruses other than influenza viruses, respiratory syncytial virus, and human metapneumovirus [7, 8]. While the development of cheaper and more user-friendly platforms will always be welcome, there needs to be a change in emphasis away from simply detecting specific organisms in a given specimen to also demonstrating that those organisms are actually causing pneumonia. The broader approach of next generation sequencing may provide novel insights into pneumonia aetiology, but has yet to show added advantage as a diagnostic tool [9].

A major recent revelation has been recognition of the lung microbiome [10]. Until recently, the lungs in health were regarded as sterile. The use of modern culture-independent techniques has not supported this notion, consistently finding evidence of bacteria in the lower airways [10]. This important realisation, along with the increasing recognition that bacteria and viruses frequently interact in the causative pathway to pneumonia [11], has challenged our traditional paradigm of pneumonia pathogenesis. In the new paradigm, dominant species emerge from the lung ecosystem in pneumonia through uncertain mechanisms, polymicrobial pneumonia may be common [12], and the bacterial versus viral pneumonia concept is naïve and unsophisticated. There is still much to understand, but there is a clear indication that new diagnostic tools for pneumonia should not just be focused on detection of specific known pathogens.

No major new innovative approaches to pneumonia diagnostics have appeared since 2009. The potential for breath analysis has still not been realised and is the subject of ongoing investigation by many research groups [13, 14]. There have also been no major new breakthroughs in the biomarker world to aid the aetiologic diagnosis of pneumonia. While there is some evidence that antibiotic management of pneumonia may be guided by blood procalcitonin levels [15], it is unclear whether procalcitonin is functioning as a marker for bacterial pneumonia or as a marker of disease severity. This is particularly pertinent as we move away from the simplistic bacterial versus viral pneumonia paradigm. It is possible that the answer may lie with certain combinations of protein biomarkers that may have greater utility for determining microbial aetiology in pneumonia [16], or that biomarkers are better used for purposes other than for determining the cause of pneumonia [17].

The findings of one of the world’s largest pneumonia aetiology studies will be published in 2016 with great anticipation. The Pneumonia Etiology Research for Child Health (PERCH) project is a seven-country case-control study, which is focused on the causes of severe pneumonia in young children from developing countries [18]. There is great interest in PERCH’s experience deploying state of the art diagnostics at each study site. However, one of the most novel aspects of PERCH is the use of sophisticated statistical modelling techniques to help interpret diagnostic test results. Partially latent class models have been designed for estimating the population aetiology distribution and the individual aetiology probabilities for specific pneumonia pathogens [19]. This approach endeavours to overcome the known limitations of diagnostic testing, and has never before been so extensively applied to the aetiology of an infectious disease. It remains to be seen what reception this approach will receive from the scientific community.

For the diagnostic laboratory, there have been only modest changes in the approach to determining the causative pathogens of pneumonia over recent years. However, we have entered a new age that could well increase the pace of development. By opening our minds to new paradigms of pneumonia pathogenesis and increasing our understanding of the lung microbiome in health and disease, additional diagnostic tools are likely to appear that will help better guide the management of pneumonia.
